# Effect of the Early Administration of Selective Serotonin Reuptake Inhibitors on the Time Course of Poststroke Fatigue: A 2-Year Longitudinal Study

**DOI:** 10.3389/fneur.2021.748473

**Published:** 2022-01-20

**Authors:** Jinjing Wang, Fang Wang, Mengmeng Gu, Lulu Xiao, Pengfei Xu, Jianglong Guo, Shiyi Jiang, Yujing Liu, Yuanlu Liu, Wen Sun, Xinfeng Liu

**Affiliations:** ^1^Department of Neurology, Affiliated Jinling Hospital, Medical School of Nanjing University, Nanjing, China; ^2^Department of Neurology, Nanjing First Hospital, Nanjing Medical University, Nanjing, China; ^3^Division of Life Sciences and Medicine, Stroke Center and Department of Neurology, The First Affiliated Hospital of USTC, University of Science and Technology of China, Hefei, China; ^4^Department of Neurology, Jinling Hospital, Nanjing Medical University, Nanjing, China; ^5^Department of Neurology, Jinling Hospital, The First School of Clinical Medicine, Southern Medical University, Nanjing, China

**Keywords:** poststroke fatigue, treatment, time course, depression, antidepressant

## Abstract

**Background:**

Poststroke fatigue (PSF) is a dynamic process over time. Current evidence for interventions for PSF is limited. Our study investigated the effects of selective serotonin reuptake inhibitors (SSRIs) on the time course of PSF.

**Methods:**

The patients with acute ischaemic stroke were enrolled in this study. All patients were scored with the Fatigue Severity Scale (FSS) at baseline and 6, 12, and 24 months after the index stroke. The time course of PSF was classified as non-PSF, incident PSF, recovered PSF and persistent PSF according to the PSF status at baseline and the 24-month follow-up. Multivariate logistic regression analysis and generalized mixed model were applied to explore the relationships between SSRIs and the time course of PSF.

**Results:**

Eight hundred forty-seven patients were analyzed in this study. No significant association between SSRIs and the time course of PSF was observed in all patients (*p* = 0.076). The subgroup analyses indicated that SSRI antidepressants reduced the risk of incident PSF [Odds Ratio (OR) 0.23; 95% confidence interval (CI) 0.05–0.96, *p* = 0.044] and persistent PSF (OR 0.28; 95% CI 0.09–0.90, *p* = 0.033) in the PSD subgroup, but not in the non-PSD group. In the longitudinal analysis, generalized logistical mixed-effect models indicated that early administration of SSRIs reduced the risk (OR 0.955; 95% CI 0.928–0.984, *p* = 0.002) and severity (β = −0.018, *p* = 0.041) of PSF in the PSD subgroup.

**Conclusion:**

No relationship was identified between the use of SSRIs and the time course of PSF in all patients. However, these drugs might be effective in PSF patients with depression.

## Introduction

Poststroke fatigue (PSF) is a common and debilitating symptom experienced by patients with stroke, with morbidity ranging from 25 to 85% due to different PSF definitions, assessment methods and the selection of population samples (1–3). PSF affects the rehabilitation and long-term outcomes of stroke survivors (4, 5). It has been reported to be a determinant of resuming work and recovering from physical disability (6, 7). However, to date, evidence on the treatment of PSF is rare, particularly pharmacological interventions (8).

Selective serotonin reuptake inhibitors (SSRIs), a commonly prescribed drug used to treat depression, have recently attracted attention as a treatment for patients with PSF. However, according to some researchers (9), fluoxetine is not effective at reducing symptoms of PSF. Similarly, another study (10) indicated that patients with acute ischaemic stroke (AIS) treated with duloxetine displayed a numerically slight improvement in fatigue, but the result was not statistically significant. The explanation for these negative results might be attributed to the short-term follow-up and small sample size, which might have underpowered some of the statistical assessments. Given the undoubted effectiveness of SSRIs in the treatment of poststroke depression (PSD) and fatigue is the core symptom of depression (11, 12), we hypothesized that SSRIs might be able to treat or prevent PSF in the subgroup of patients with PSD. Hence, our aim was to explore the effects of SSRIs on PSF in a large observational cohort with 2 years of follow-up. In addition, we adopted the method of a stratified analysis to determine the effects of SSRIs on PSF in the PSD subgroup.

## Methods

### Study Population

This study was a retrospective review of a prospective database, which was conducted in a large-scale general hospital in China from October 2015 to May 2017 with 2 years of follow-up (13). The patients were diagnosed with acute ischaemic stroke and administered standard antithrombotic treatment according to the guidelines and recommendations (14). The other treatment options, particularly antidepressants (mainly SSRIs), prescribed not only to treat/prevent PSD or poststroke anxiety but also to potentially accelerate rehabilitation (15, 16), were based on the clinicians' choices and patients' willingness. The inclusion criteria for this study were (1) age ≥18 years, (2) diagnosed with AIS by magnetic resonance imaging, and (3) ability and willingness to participate in this study. The exclusion criteria included (1) patients with a communication disorder who were unable to provide a reliable interview due to unconsciousness, severe aphasia, dementia and other conditions; (2) patients with other known causes and neuropsychiatric disease (e.g., multiple sclerosis, Parkinson's disease, malignancies, severe heart failure or severe sleep disorder); (3) regular use of sleeping pills or other medications that cause changes in the emotional status (e.g., sedatives, antidepressants, antipsychotic drugs, antiepileptic drugs or glucocorticoids); (4) presence of depression or fatigue before the index stroke; (5) treatment with other types of antidepressants (e.g., serotonin and norepinephrine reuptake inhibitors, tricyclic antidepressants, atypical antidepressants and other medications), including monotherapy or combination therapy; and (6) recurrent stroke or TIA, death and neurological deterioration during follow-up. Moreover, all patients were assessed with the Mini-Mental State Examination (MMSE) and those with MMSE score ≤ 10 or MMSE score 11 to 23 but found cognitively incompetent were excluded.

The patients who took SSRIs for at least 1 month after the index stroke were categorized as the treated group. Patients who did not take or took SSRIs for <1 month were regarded as the untreated group.

We received approval from the local Ethics Committee on Human Experimentation. Informed consent was obtained from all participants.

### Data Recorded

Demographic characteristics, vascular risk factors, and neurological function scores were collected. The following specific variables were recorded: baseline characteristics, such as age, sex and body mass index (BMI); vascular risk factors [diabetes mellitus (DM), hypertension, smoking, drinking, and hyperlipemia]; and medical history [previous stroke/TIA and atrial fibrillation (AF)]. BMI was defined as the weight in kilograms divided by the square of the height in meters. Stroke severity was assessed by calculating the NIH Stroke Scale (NIHSS) score. The stroke subtype was classified according to the Trial of Org 10172 in Acute Stroke Treatment (TOAST) criteria (17). Neurological status at discharge was assessed with the modified Rankin Scale (mRS).

### Assessments of Fatigue and Other Related Parameters

All patients were assessed with the Chinese version of the Fatigue Severity Scale (FSS), a 7-point Likert scale ranging from strongly disagree to strongly agree (18, 19). Patients with a mean FSS score of four points or more were considered to have fatigue. Poststroke depression was diagnosed according to the criteria of the Diagnostic and Statistical Manual (DSM-V) (20). The symptoms of depression, anxiety and social interpersonal relationships were assessed using the 24-item Hamilton Depression Scale (HAMD-24) (21), 14-item Hamilton Anxiety Scale (HAMA-14) (22) and Lubben Social Network Scale (LSNS) (23), respectively. Higher scores indicated more severe depression and anxiety and better interpersonal communication.

### Follow-Up Assessments

All included patients completed the 2-year follow-up, which included no <3 time points after the index stroke (6, 12, and 24 months). The severity of fatigue was assessed with the FSS during follow-up. The patients were divided into four groups according to the PSF status at baseline and the time point of the 24th month after the index stroke. The patients were classified as follows: non-PSF (patients without fatigue at baseline and 24 months); incident PSF (patients without fatigue at baseline but with fatigue 24 months); recovered PSF (patients with fatigue at baseline but without fatigue at 24 months); and persistent fatigue (patients with fatigue at baseline and 24 months).

### Statistical Analysis

Continuous variables are presented as the means ± SD or medians (interquartile ranges), and categorical variables are presented as numbers (percentages). Categorical variables were compared with the χ^2^ test, and continuous variables were compared with Student's *t* test or the Mann-Whitney U test, as appropriate. Univariate analysis was conducted to compare baseline characteristics between the fatigue group and the non-fatigue group in the acute phase. A propensity score matching (PSM) analysis was used performed to balance heterogeneity between the treated group and the untreated group. Variables used for calculating the propensity score (PS) included age, sex, BMI, hypertension, DM, hyperlipemia, AF, smoking, drinking, previous stroke and TOAST classification. The 1:1 nearest-neighbor matching algorithm was applied to create the matched treated group and untreated group with a caliper distance of 0.1.

Furthermore, multivariable logistic regression analyses were conducted to evaluate the association between SSRIs and the time course of PSF in subgroups of patients with and without PSD at baseline. Multivariate generalized linear and logistic mixed-effect models were performed to show the association between SSRIs and multidomain longitudinal PSF obtained at baseline and at 6, 12, and 24 months. The first-order autoregression (AR1) model was selected as the suitable model based on the Akaike information criteria (AIC) for all models. The analysis was performed using SPSS statistics 26.0 software (IBM Corp, Armonk, NY) and Stata/SE 15.0 (StataCorp LP, College Station TX).

Two-sided values of p < 0.05 were considered statistically significant. The Bonferroni correction method was applied to adjust the *p* values for multiple comparisons.

## Results

### Baseline Characteristics

After applying the eligibility criteria, 847 of the 1,260 screened patients completed the 24-month follow-up and were included in the study. Nine of these patients did not respond at the 6 month follow-up, 10 patients were deciduous at the 12-month follow-up, and 2 patients were unable to be contacted at the 6- and 12-month follow-ups, but they all re-entered the study at the 24-month follow-up. The prevalence of PSF was 41.6% in the acute phase, and the mean age was 60.6 ± 13.1 years. Six hundred fourteen (72.5%) of the patients were male. A comparison of the baseline characteristics between patients with and without fatigue at study entry are shown in Supplementary Table e-1.

Three hundred nine (36.5%) patients were treated with SSRI antidepressants, including escitalopram (87%), citalopram (4%), sertraline (6%) and paroxetine (3%). Compared with untreated group, the HAMD score [3(1, 5) vs. 8 (2,14), *p* < 0.001] and HAMA score [2(0, 5) vs. 4(1, 9), *p* < 0.001] of the treated group were higher, and the treated group was more likely to be male (75.5 vs. 67.3%, *p* = 0.011), had a higher proportion of patients with PSD (57.9 vs. 13%, *p* < 0.001) and a lower proportion of patients with hyperlipemia (17.8 vs. 24.0%, *p* = 0.032). After the 1:1 PSM analysis, data from each of the 304 patients in the treated group and the untreated group were extracted. The flowchart for the selection is displayed in Supplementary Figure e-1. The baseline significant differences in sex and hyperlipemia between the treated group and the untreated group were reduced after applying the PSM method. Details of the characteristics before and after PSM are shown in Table 1.

**Table 1:** Comparison of the baseline characteristics and neuropsychological scores between unmatched and propensity score-matched groups.

	**Unmatched**		***P* value**	**Matched**		***P* value**
	**Untreated**	**Treated**		**Untreated**	**Treated**	
	**(n=538)**	**(n=309)**		**(*n* = 304)**	**(*n* = 304)**	
Age, mean (SD), y	60.3 ± 13.4	61.1 ± 12.5	0.548	61.2 ± 14.1	61.1 ± 12.5	0.932
Male, *n* (%)	406 (75.5)	208 (67.3)	0.011	204 (67.1)	207 (68.1)	0.664
BMI, mean (SD), kg/m^2^	24.72 ± 3.08	24.75 ± 3.27	0.900	24.69 ± 3.21	24.75 ± 3.28	0.815
Hypertension, *n* (%)	388 (72.1)	236 (76.4)	0.176	227 (74.7)	232 (76.3)	0.637
DM, *n* (%)	191 (35.5)	93 (30.1)	0.109	98 (32.2)	92 (30.3)	0.600
Hyperlipemia, *n* (%)	108 (20.1)	81 (26.2)	0.039	60 (19.7)	69 (22.7)	0.372
CHD, *n* (%)	50 (9.3)	34 (11.0)	0.423	28 (9.2)	32 (10.5)	0.586
Smoking, *n* (%)	189 (35.1)	102 (33.0)	0.532	104 (34.2)	102 (33.6)	0.864
Drinking, *n* (%)	125 (23.2)	79 (25.6)	0.445	73 (24.0)	77 (25.3)	0.707
Stroke, *n* (%)	159 (29.6)	73 (23.6)	0.063	66 (21.7)	72 (23.7)	0.561
TOAST, *n* (%)			0.733			0.708
LAA	216 (40.1)	124 (40.1)		117 (38.5)	123 (40.5)	
SAD	210 (39.0)	127 (41.1)		122 (40.1)	124 (40.8)	
Others^a^	112 (20.8)	58 (18.8)		65 (21.4)	57 (18.8)	
NIHSS, median (IQR)	2 (1–6)	2 (0–4)	0.336	2 (1–7)	2 (0–5)	0.099
mRS, median (IQR)	1 (0–2)	1 (0–2)	0.927	1 (0–2)	1 (0–2)	0.521
HAMD, median (IQR)	3 (1–5)	8 (2–14)	<0.001	3 (1–5)	8 (2–14)	<0.001
HAMA, median (IQR)	2 (0–5)	4 (1–9)	<0.001	2 (0–5)	4 (1–9)	<0.001
LUBBEN, median (IQR)	32 (23–40)	32 (24–38)	0.184	32 (19–40)	32 (24–37)	0.249
PSD, *n* (%)	70 (13.0)	179 (57.9)	<0.001	42 (13.8)	177 (58.2)	<0.001
24 month-PSF, *n* (%)	134 (24.9)	73 (23.6)	0.676	77 (25.3)	71 (23.4)	0.772
Time course of PSF, *n* (%)			0.154			0.076
Non-PSF	281 (52.2)	150 (48.5)		161 (53.0)	147 (48.4)	
Incident PSF	46 (8.6)	17 (5.5)		28 (9.2)	16 (5.3)	
Recovered PSF	123 (22.9)	86 (27.8)		66 (21.7)	86 (28.3)	
Persistent PSF	88 (16.4)	56 (18.1)		49 (16.1)	55 (18.1)	

*BMI, body mass index; CHD, coronary heart disease; DM, diabetes mellitus; HAMA, Hamilton Anxiety Scale; HAMD, Hamilton Depression Scale; IQR, interquartile range; LAA, large artery atherosclerosis; mRS, modified Rankin Scale; NIHSS, NIH Stroke Scale; PSD, poststroke depression; PSF, poststroke fatigue; SAD, small artery occlusion; SD, standard deviation; TOAST, Trial of Org 10172 in Acute Stroke Treatment*.

a *Others, cardioembolism, stroke of other determined cause, and stroke of undetermined cause*.

*Variables used for calculating the propensity score (PS) included age, sex, BMI, hypertension, DM, hyperlipemia, AF, smoking, drinking, previous stroke and TOAST classification*.

### Effect of SSRIs on the Time Course of PSF in the Overall Population

In the final analysis, the time course of PSF was stratified into four groups (non-PSF, incident PSF, recovered PSF and persistent PSF) according to the aforementioned criteria. The proportions of the 4 groups of patients were 50.9, 7.4, 24.7 and 17%, respectively. No significant difference in different time course of PSF was observed between the treated group and untreated group both in the unmatched data (*p* = 0.154) and PSM (*p* = 0.076) analysis (Table 1). The multivariate analysis showed that SSRI antidepressants did not significantly reduce the risk of incident PSF (OR 0.93; 95% CI 0.49–1.76, *p* = 0.815) or persistent PSF (OR 1.02; 95% CI 0.60–1.74, *p* = 0.944) compared with the non-PSF group and the recovered PSF group after adjusting for age, sex, hypertension, DM, AF, smoking, drinking, history of stroke or TIA, dyslipidaemia, TOAST classification, and NIHSS, mRS, HAMA and LSNS scores.

### Effect of SSRIs on the Time Course of PSF in the Subgroup Analysis

The patients were divided into the PSD group and the non-PSD group according to the prespecified analysis. In the non-PSD subgroup, the distribution of the time course of PSF within the four groups was not significantly different (Supplementary Figure e-2a). However, in the PSD subgroup, the distribution of these groups was significantly different (*p* < 0.001). An intragroup analysis indicated an increased proportion of patients with recovered PSF (*p* < 0.001) and a decreased proportion of patients with persistent PSF (*p* < 0.001) compared with untreated patients (Supplementary Figure e-2b). Multivariable adjustments showed that SSRI antidepressants significantly reduced the risk of incident PSF (OR 0.23; 95% CI 0.05–0.96, *p* = 0.044) and persistent PSF (OR 0.28; 95% CI 0.09–0.90, *p* = 0.033) in the PSD subgroup. However, for the non-PSD subjects, the correlation was not statistically significant (Table 2).

**Table 2:** Multivariate logistic regression analysis of the association of SSRIs with time course of PSF.

	**All patients (*****n*** **=** **847)**		**Non-PSD patients (*****n*** **=** **598)**		**PSD patients (*****n*** **=** **249)**	
	**OR (95% CI)**	***P* value**	**OR (95% CI)**	***P* value**	**OR (95% CI)**	***P* value**
Non-PSF (*n* = 431)	reference		reference		reference	
Incident PSF (*n* = 63)	0.93 (0.49–1.76)	0.815	1.33 (0.57–3.08)	0.507	0.23 (0.05–0.96)	0.044
Recovered PSF (*n* = 209)	reference		reference		reference	
Persistent PSF (*n* = 144)	1.02 (0.60–1.74)	0.944	0.97 (0.38–2.46)	0.940	0.28 (0.09–0.90)	0.033

*The model was adjusted for age, sex, body mass index, hypertension, diabetes mellitus, hyperlipemia, smoking, drinking, TOAST classification, NIHSS, HAMA and Lubben score*.

*CI, confidence interval; HAMA, Hamilton Anxiety Scale; NIHSS, NIH Stroke Scale; OR, odds ration; PSD, poststroke depression; PSF, poststroke fatigue; TOAST, Trial of Org 10172 in Acute Stroke Treatment*.

### Longitudinal Analysis of the Effect of SSRIs on PSF

**Figure 1:**
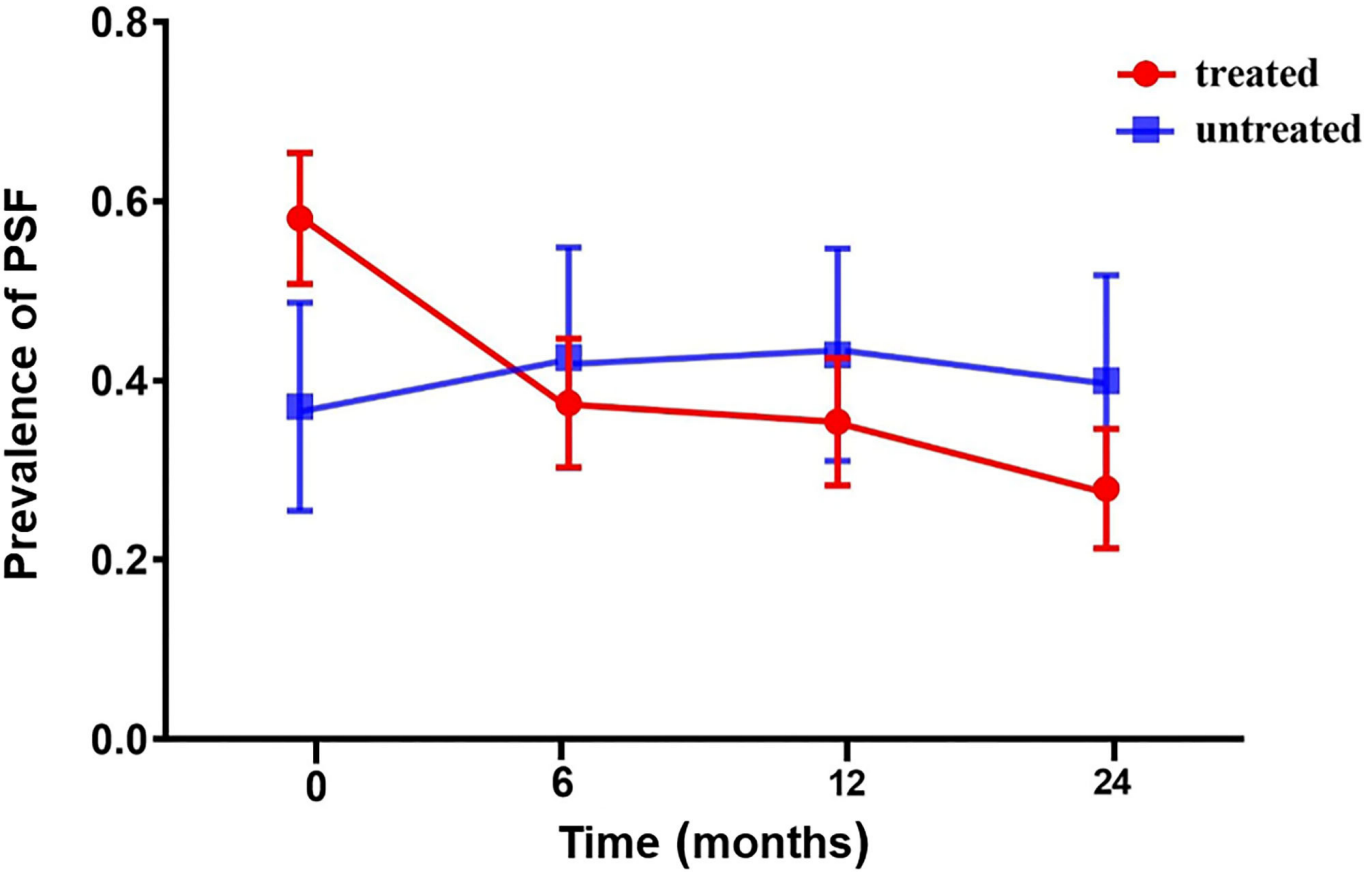
The prevalence of PSF in the acute phase and at 6, 12, and 24 months of follow-up in the PSD subgroup. Compared with untreated patients, the incidence of PSF in patients treated with SSRIs was higher at baseline. The incidence rate of patients in the treated group decreased over time (red line). The incidence rate of the untreated group increased over the first 6 months and then decreased (blue line). PSD, poststroke depression; PSF, poststroke fatigue.

Figure 1 shows a decrease in the prevalence of PSF over time in the PSD subgroup, particularly in the treated group. Generalized logistic mixed-effect models indicated that SSRIs reduced the risk of PSF in the PSD subgroup (OR 0.952; 95% CI 0.928–0.976; *p* < 0.001) at each follow-up time point. Multivariate adjustments did not significantly alter the results (OR 0.955; 95% CI 0.928–0.984; *p* = 0.002; Table 3).

**Table 3:** Generalized logistical mixed-effect model of the relationship between SSRIs and PSF during the 24-month follow-up period.

	**Model 1**		**Model 2**	
	**OR (95% CI)**	***P* value**	**OR (95% CI)**	***P* value**
**All patients (*****n*** **=** **847)**
Treatment	0.837 (0.694–1.011)	0.064	0.991 (0.976–1.007)	0.343
Time	0.688 (0.615–0.769)	<0.001	0.970 (0.961–0.980)	<0.001
**PSD group (*****n*** **=** **249)**
Treatment	0.952 (0.928–0.976)	<0.001	0.955 (0.928–0.984)	0.002
Time	1.001 (0.981–1.022)	0.903	0.997 (0.973–1.022)	0.823
**Non-PSD group (*****n*** **=** **598)**
Treatment	1.029 (0.790–1.338)	0.834	1.008 (0.984–1.033)	0.520
Time	0.658 (0.587–0.738)	<0.001	0.965 (0.954–0.975)	<0.001

*Model 1: Unadjusted; Model 2: The model was adjusted for age, sex, body mass index, hypertension, diabetes mellitus, hyperlipemia, smoking, drinking, TOAST classification., NIHSS, HAMA and Lubben score*.

*CI, confidence interval; HAMA, Hamilton Anxiety Scale; NIHSS, NIH Stroke Scale; TOAST, Trial of Org 10172 in Acute Stroke Treatment; OR, odds ration; PSD, poststroke depression; PSF, poststroke fatigue*.

Generalized linear mixed-effect models of the PSD subgroup indicated that the severity of PSF was reduced from baseline to 24 months after stroke onset with time as the main effect (β = −0.034, *p* < 0.001). Moreover, the FSS score of the treated group showed a significantly greater decrease than the untreated group (β = −0.016, *p* = 0.021). Multivariate adjustments remained significant (β = −0.018, *p* = 0.041; Table 4).

**Table 4:** Multiple linear mixed-effect regression analysis of the relationship between SSRIs and PSF in the PSD subgroup during the 24-month follow-up period.

	**β (95% CI)**	**SE**	***t* value**	***P* value**
**Model 1**
Baseline FSS	0.165 (−0.188–0.519)	0.180	0.92	0.359
Treatment	−0.016 (−0.030–0.002)	0.007	−2.313	0.021
Time effect	−0.035 (−0.047–0.024)	0.006	−5.94	<0.001
**Model 2**
Baseline FSS	0.001 (−0.383–0.386)	0.196	0.008	0.994
Treatment	−0.018 (−0.035–0.001)	0.009	−2.05	0.041
Time effect	−0.034 (−0.0495–0.020)	0.007	−4.69	<0.001

*Model 1: Unadjusted; Model 2: The model was adjusted for age, sex, body mass index, hypertension, diabetes mellitus, hyperlipemia, smoking, drinking, TOAST classification., NIHSS, HAMA and Lubben score*.

*CI, confidence interval; CHD, coronary heart disease; HAMA, Hamilton Anxiety Scale; NIHSS, NIH Stroke Scale; TOAST, Trial of Org 10172 in Acute Stroke Treatment; PSD, poststroke depression; PSF, poststroke fatigue; SE, standard error*.

However, for the overall subjects and patients in the non-PSD subgroup, we failed to detect statistically significant associations between SSRIs and the risk or severity of PSF (Table 3 and Supplementary Tables e-2a and e-2b).

## Discussion

Based on the present study, SSRI antidepressants did not exert a significant effect on the time course of fatigue in all patients with AIS. However, in the subgroup of patients with PSD, the use of SSRI antidepressants potentially reduced the risks of incident PSF and persistent PSF. In the longitudinal analysis of the PSD subgroup, the risk and severity of PSF was lower in treated patients than in untreated patients.

The purpose of this study was to investigate the association between SSRI antidepressants and fatigue in patients with AIS. The results did not reveal a significant association between SSRI antidepressants and the time course of PSF. This finding was consistent with previous studies (9, 10) showing that fluoxetine, duloxetine, citalopram and sertraline were not effective at relieving PSF. This negative result might be explained by the fact that fatigue is a multidimensional constellation of symptoms; therefore, treatment with a single SSRI antidepressant might be an ineffective strategy for all patients with PSF (24).

PSF is independent of depression in patients with stroke, as fatigue symptoms are common in non-depressed patients (25). However, the existence of depression aggravates the feeling of fatigue (26). Based on the results of the multivariate analysis and longitudinal cohort analysis in this study, SSRIs reduced the long-term risk and severity of PSF. Early administration of antidepressants might be effective at improving fatigue symptoms in patients with depression because early improvement of mood in patients with depression may contribute to better treatment compliance and active participation in daily rehabilitation training (27). The incidence of late fatigue will decrease when patients with stroke have a positive mood and good functional status (28). One possible speculation is that brain serotonin and dopamine levels play a leading role in the development of fatigue in patients with depression, and thus SSRI antidepressant drugs may effectively relieve the symptoms of fatigue in patients with PSD (29). Another explanation for this finding is that common inflammatory mechanisms between PSD and PSF might exist because depression and fatigue are both considered inflammation-related diseases. Thus, SSRI treatment for PSD may reduce the levels of inflammatory cytokines related to PSF to relieve fatigue symptoms (30).

## Limitations

The strengths of this study include the large number of participants, the long duration of follow-up, and the relatively detailed analysis. However, several limitations should be considered. First, this study has all the typical limitations inherent in any single-center observational analysis, although we used the PSM method to control the heterogeneity between the treated group and the untreated group. Second, we did not analyse the differences between specific drugs because the number of patients treated with different SSRI drugs varied substantially. Third, we excluded patients with higher NIHSS scores to complete the scale assessment successfully, which may lead to selection bias and may not represent all patients with AIS. Fourth, we did not regularly monitor whether the patient took SSRIs in the late course of stroke but not in the acute phase, which might underestimate the effects of SSRIs. Fifth, although the total sample size was large, some subgroups were small, yielding a limited power for the statistical analyses. Sixth, some missing information prevented us from completing the analysis of time and dose dependence of the drug, which limits generalization of the results. Finally, we excluded patients treated with non-SSRI antidepressants to avoid the interaction of different antidepressants with different mechanisms, which might also lead to a potential underestimation of the effects of SSRIs.

## Conclusion

In conclusion, SSRI antidepressants had little effect on long-term PSF but resulted in a significant improvement in the therapeutic and preventive effects on patients with PSF presenting with depression. Further multicentre, double-blind, randomized controlled trials are needed to prove the effectiveness of pharmacological therapy and related mechanisms of PSF.

## Data Availability Statement

The original contributions presented in the study are included in the article/Supplementary Material, further inquiries can be directed to the corresponding authors.

## Ethics Statement

The studies involving human participants were reviewed and approved by the Ethics Committee of Jinling Hospital. The patients/participants provided their written informed consent to participate in this study.

## Author Contributions

JW, FW, and MG contributed to the concept and design of the study. JW conducted the data analysis and wrote the first draft of the manuscript. LX and PX conducted the data analysis. JG, YujL, SJ, and YuaL contributed to data collection. WS and XL contributed to the study design, interpretation of results, and critical revision of manuscript. All authors contributed to the article and approved the submitted version.

## Funding

This work was supported by the National Science Foundation of China (81870946 and 81701184), Anhui Provincial Natural Science Foundation (2008085QH368 and 2108085MH271), Key Research and Development Plan Projects of Anhui Province (202104j07020049) and Jiangsu Provincial Outstanding Medical Talented Project (JCRCB2016005) and the Fundamental Research Funds for the Central Universities.

## Conflict of Interest

The authors declare that the research was conducted in the absence of any commercial or financial relationships that could be construed as a potential conflict of interest.

## Publisher's Note

All claims expressed in this article are solely those of the authors and do not necessarily represent those of their affiliated organizations, or those of the publisher, the editors and the reviewers. Any product that may be evaluated in this article, or claim that may be made by its manufacturer, is not guaranteed or endorsed by the publisher.
