# Supplementary material for: Effect of the Early Administration of Selective Serotonin Reuptake Inhibitors on the Time Course of Poststroke Fatigue: A 2-Year Longitudinal Study
Source: Front Neurol. 2022 Jan 20;12:748473. doi: 10.3389/fneur.2021.748473 (PMC8811294; doi:10.3389/fneur.2021.748473)
Supplement: Supplementary file 1 [file Data_Sheet_1.docx]

Table e-1 baseline characteristics between without PSF and PSF in the acute phase

|  | Without PSF (n=494) | With PSF (n=353) |
| --- | --- | --- |
| Age, mean (SD), y | 60.0 ± 12.4 | 61.4 ± 13.9 |
| Male, n (%) | 370 (74.9) | 244 (69.1) |
| BMI, mean (SD), kg/m^2^ | 24.91 ± 3.17 | 24.49 ± 3.11 |
| Hypertension, n (%) | 358 (72.5) | 266 (75.4) |
| AF, n (%) | 145 (29.4)** | 139 (39.4)** |
| Hyperlipemia, n (%) | 97 (19.6) | 72 (26.1) |
| CHD, n (%) | 48 (9.7) | 36 (10.2) |
| Smoking, n (%) | 16 (33.6) | 125 (35.4) |
| Drinking, n (%) | 116 (23.5) | 88 (24.9) |
| Stroke, n (%) | 125 (25.3) | 107 (30.3) |
| TOAST classification, n (%) |  |  |
| LAA | 204 (41.3) | 136 (38.5) |
| SAD | 206 (41.7) | 131 (37.1) |
| Others | 84 (17.0)* | 86 (24.4)* |
| SSRI, n (%) | 167 (33.8) | 142 (40.2) |
| PSD, n (%) | 119 (24.1)*** | 130 (36.8)*** |
| Later PSF, n (%) | 63 (12.8)*** | 144 (40.8)*** |
| HAMA, n (%) | 3 (0-5)** | 4 (1-7)** |
| LSNS, n (%) | 32 (20-40) | 32 (24.5-37) |
| NIHSS on admission, median(IQR) | 3 (1-7)** | 2 (0-4)** |
| MRS after at discharge, median(IQR) | 1 (0-2)* | 1 (0-1.3)* |

Abbreviations: AF=atrial fibrillation; BMI=body mass index; DM = diabetes mellitus; HAMA=Hamilton Anxiety Rating Scale; HAMD=Hamilton Depression Scale; LAA=large artery atherosclerosis; LSNS= lubben social network scale; MRS= modified ranking scale; NIHSS=National Institute of Health Stroke Scale; Others=Cardioembolism, Stroke of other determined cause and Stroke of undetermined cause; PSD=poststroke depression; PSF=poststroke fatigue; SAD=small artery occlusion; SSRI= selective serotonin reuptake inhibitor; TOAST = Trial of Org10172 in Acute Stroke Treatment.*p<0.05, **p<0.01,***p<0.001.

Table e-2a Multiple linear mixed-effect regression between SSRI and PSF during 24-month follow-up in all patients

|  |  |  |  |  |
| --- | --- | --- | --- | --- |
|  | β (95%CI) | SE | t value | p value |
| model1 |  |  |  |  |
| baseline FSS | 0.120 (-0.067 to 0.306) | 0.095 | 1.26 | 0.208 |
| treatment | -0.006 (-0.014 to 0.002) | 0.004 | -1.38 | 0.167 |
| time effect | -0.037 (-0.042 to -0.032) | 0.003 | -14.77 | <0.001 |
| model2 |  |  |  |  |
| baseline FSS | 0.096 (-0.098 to 0.289) | 0.099 | 0.969 | 0.333 |
| treatment | -0.004 (-0.013 to 0.004) | 0.004 | -1.029 | 0.304 |
| time effect | 0.037 (-0.042 to -0.032) | 0.003 | -14.113 | <0.001 |

Model 1: unadjusted; Model 2: adjusted for age, sex, body mass index, hypertension, diabetes mellitus, hyperlipemia, atrial fibrillation, smoking, drinking, TOAST classification., NIHSS, HAMA and Lubben score. CI, confidence interval; CHD, coronary heart disease; HAMA, Hamilton Anxiety Scale; NIHSS, NIH Stroke Scale; TOAST, Trial of Org 10172 in Acute Stroke Treatment; PSD, poststroke depression; PSF, poststroke fatigue. SE, standard error.

Table e-2b Multiple linear mixed-effect regression between SSRI and PSF during 24-month follow-up in the non-PSD subgroup

|  | β (95%CI) | SE | t value | p value |
| --- | --- | --- | --- | --- |
| model1 |  |  |  |  |
| intercept | 3.181 (3.061 to 3.300) | 0.061 | 52.09 | <0.001 |
| baseline FSS | -0.302 (-0.559 to -0.045) | 0.131 | -2.304 | 0.021 |
| treatment | 0.003 (-0.008 to 0.014) | 0.006 | 0.495 | 0.621 |
| time effect | -0.037 (-0.043 to -0.032) | 0.003 | -14.15 | <0.001 |
| model2 |  |  |  |  |
| intercept | 3.167 (2.549 to 3.785) | 0.315 | 10.04 | <0.001 |
| baseline FSS | -0.224 (-0.485 to 0.037) | 0.133 | -1.68 | 0.093 |
| treatment | 0.003 (-0.009 to 0.015) | 0.006 | 0.499 | 0.618 |
| time effect | -0.038 (-0.043 to -0.032) | 0.003 | -13.40 | <0.001 |

Model 1: unadjusted; Model 2: adjusted for age, sex, body mass index, hypertension, diabetes mellitus, hyperlipemia, atrial fibrillation, smoking, drinking, TOAST classification., NIHSS, HAMA and Lubben score. CI, confidence interval; CHD, coronary heart disease; HAMA, Hamilton Anxiety Scale; NIHSS, NIH Stroke Scale; TOAST, Trial of Org 10172 in Acute Stroke Treatment; PSD, poststroke depression; PSF, poststroke fatigue. SE, standard error.

Figure e- 1 Flowchart of patients included in this study


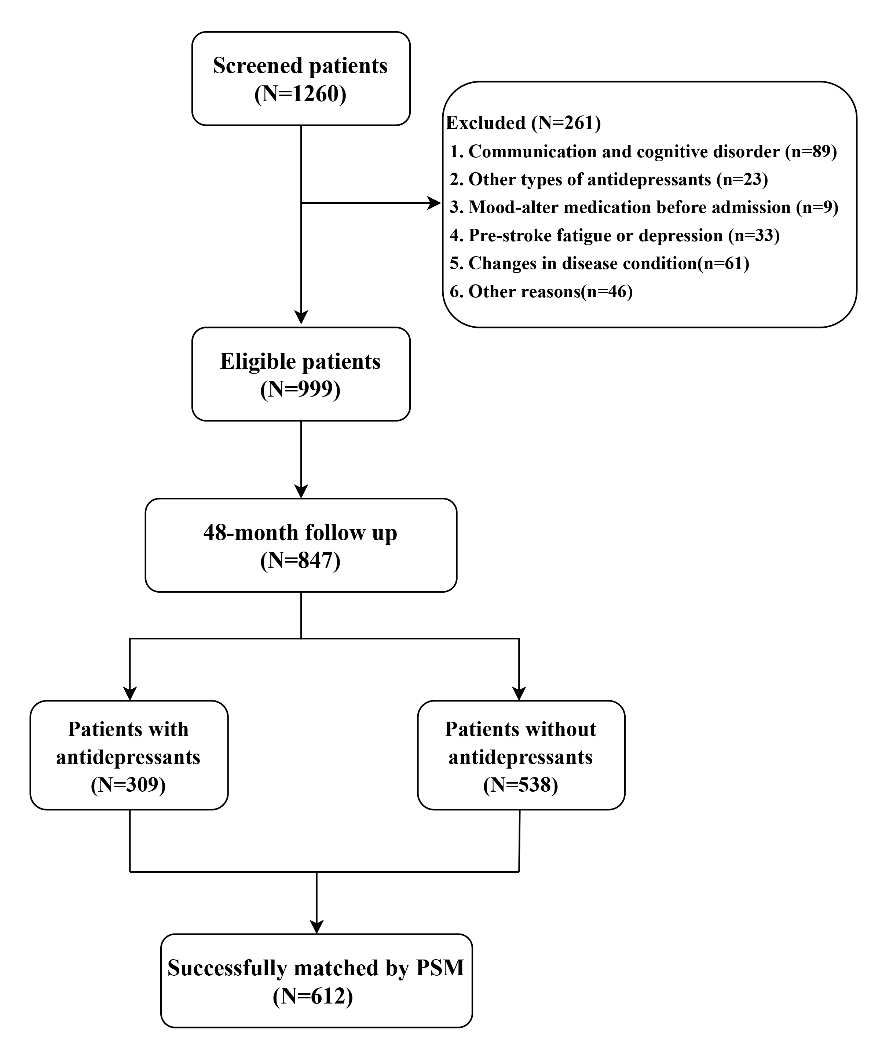


Figure e-2a


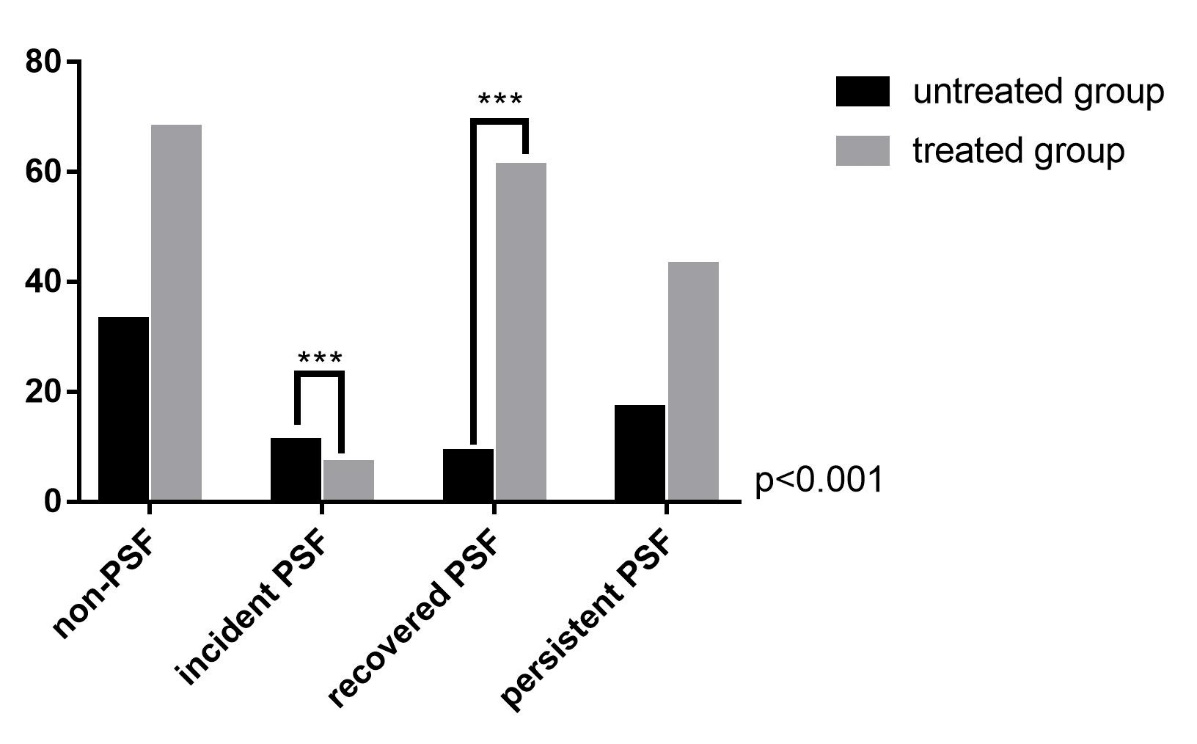


Figure e-2b


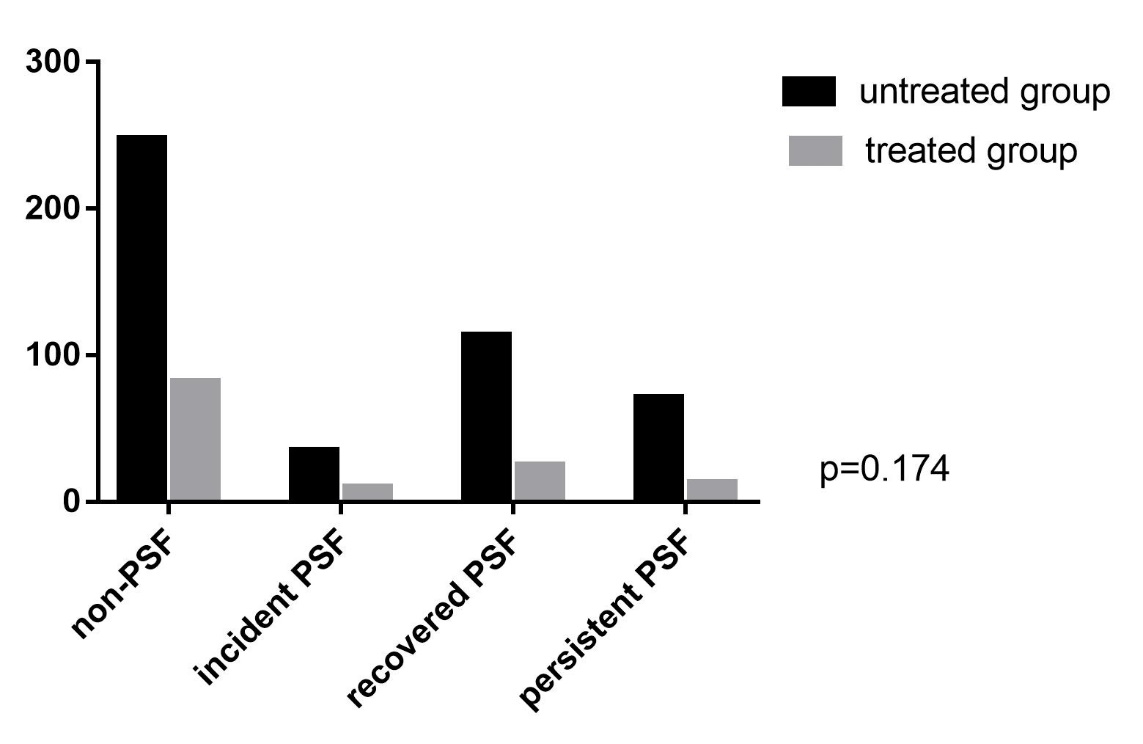


Figure e-2a shows the effects of SSRIs on the time course of PSF in the PSD subgroup. Different time courses of PSF indicated significant differences between the treated group and untreated group (p<0.001). The intragroup analysis indicated an increased proportion of patients with recovered PSF (p<0.001) and a decreased proportion of patients with persistent PSF (p<0.001) compared with untreated patients. Figure e-2b shows the effects of SSRIs on the time course of PSF in the non-PSD subgroup. The distribution of the time course of PSF within the 4 groups was not significantly different. The Bonferroni correction method was applied to multiple comparisons using a p value <0.05/no.
